# The mediating role of attachment and anger: exploring the impact of maternal early-life maltreatment on child abuse potential

**DOI:** 10.3389/fpsyt.2023.1267038

**Published:** 2023-10-27

**Authors:** Karolina Wuebken, Felix Bermpohl, Katja Boedeker, Catherine Hindi Attar, Dorothea Kluczniok, Nikola Schoofs, Anna Fuchs, Corinne Neukel, Sabine C. Herpertz, Romuald Brunner, Sibylle Maria Winter, Michael Kaess, Charlotte Jaite, Katja Dittrich

**Affiliations:** ^1^Department of Psychiatry and Psychotherapy, Charité—Universitätsmedizin Berlin, Charité Campus Mitte, Corporate Member of Freie Universität Berlin, Humboldt-Universität zu Berlin, and Berlin Institute of Health (BIH), Berlin, Germany; ^2^Adolescent Psychiatry, Psychosomatics and Psychotherapy, Charité—Universitätsmedizin Berlin, Charité Campus Virchow, Corporate Member of Freie Universität Berlin, Humboldt-Universität zu Berlin, and Berlin Institute of Health (BIH), Berlin, Germany; ^3^Department of Child and Adolescent Psychiatry, Center for Psychosocial Medicine, Heidelberg University Hospital, Heidelberg, Germany; ^4^Department of General Psychiatry, Center for Psychosocial Medicine, Heidelberg University Hospital, Heidelberg, Germany; ^5^Department of Child and Adolescent Psychiatry and Psychotherapy, University of Regensburg, Regensburg, Germany; ^6^University Hospital of Child and Adolescent Psychiatry and Psychotherapy, University of Bern, Bern, Switzerland; ^7^Department of Clinical Psychology and Psychotherapy in Childhood and Adolescence, University of Hildesheim, Hildesheim, Germany

**Keywords:** cycle of abuse, child abuse potential, attachment insecurity, anger, psychopathology

## Abstract

**Background:**

Maternal early-life maltreatment (ELM) increases the risk of subsequent child maltreatment, but the underlying mechanisms of these intergenerational effects remain largely unknown. Identifying these mechanisms is crucial for developing preventive interventions that can break the cycle of abuse. Notably, previous research has shown that ELM often results in attachment insecurity and altered anger characteristics. Therefore, this study determines whether these characteristics mediate the relationship between maternal history of ELM and child abuse potential.

**Methods:**

The study sample included 254 mothers, of whom 149 had experienced ELM to at least a moderate degree. Maternal ELM was assessed using the Childhood Experience of Care and Abuse (CECA) interview. Attachment insecurity, trait anger and anger expression, and maternal abuse potential were assessed using the Vulnerable Attachment Questionnaire (VASQ), State–Trait Anger Expression Inventory (STAXI), and Child Abuse Potential Inventory (CAPI), respectively.

**Results:**

The severity of maternal ELM predicted higher child abuse potential, with attachment insecurity and anger suppression mediating this effect. Specifically, higher levels of maternal ELM were associated with greater attachment insecurity and increased anger suppression, resulting in a higher child abuse potential. Although higher levels of trait anger were directly associated with higher child abuse potential, this parameter did not mediate the relationship with ELM. In addition, no significant associations were observed between outwardly expressed anger and ELM or child abuse potential. All analyses were adjusted for maternal mental disorders, years of education, and relationship status.

**Discussion:**

Attachment insecurity and anger suppression may serve as pathways linking the maternal history of ELM to the risk of child abuse, even when considering maternal psychopathology. Overall, our findings indicate that interventions aimed at strengthening attachment and improving anger suppression may be beneficial for all mothers with ELM history and high child abuse potential, not just those who suffer from mental illness.

## 1. Introduction

Early-life maltreatment (ELM) is a significant and widespread phenomenon. A meta-analysis estimated worldwide prevalence rates based on self-reports, revealing 36.6, 22.6, and 12.7% for emotional, physical, and sexual abuse, respectively, with 16.3 and 18.4% for physical and emotional neglect, respectively (1). Large-scale surveys conducted in Europe and Germany also reported similar overall rates of child maltreatment at 35 and 31%, respectively (2, 3).

Early-life maltreatment can have long-lasting impacts on the mental health of victims (4–6) and can also affect subsequent generations. Parents with an ELM history are at a higher risk of engaging in abusive behavior toward their children (7, 8). The prevalence and consequences of child maltreatment highlight the need for deeper insights into the intergenerational mechanisms to design and implement preventive interventions that could break the cycle of abuse. The present study determines whether the factors attachment insecurity and altered anger mediate the relationship between maternal history of ELM and child abuse potential. Attachment insecurity and altered anger were chosen because they both may result from ELM and, at the same time, be related to each other. Bowlby proposed that anger is a functional protest reaction to others negative attachment behavior and that insecure attachment may transform this functional response (anger of hope) in dysfunctional anger (anger of despair) (9). Corroborating Bowlby’s theory, previous research found an association between insecure attachment and dysfunctional anger (10, 11). Thus, investigating these two related factors may help to identify starting points for preventive measures.

While the examination of substantiated cases of maltreatment is a commonly employed research method, it may offer only a partial view of the issue due to the presence of underreporting (12, 13). Another approach involves evaluating child abuse potential through the assessment of various parental attributes that have shown associations with abusive behavior (14). Taking this approach, in our study we used the German Version of the Child Abuse Potential Inventory (CAPI) to capture a caretaker environment with a heightened risk for child abuse (15), which in itself can be detrimental to a child’s development even though actual acts of abuse do not take place (16). Although the CAPI has been shown to successfully distinguish abusive and non-abusive parents with both high sensitivity (81.4%) and specificity (99%) and to predict future official maltreatments reports effectively (17, 18), high CAPI scores do not identify an abusive parent. Accordingly, we used child abuse potential as dimensional measure for the risk of child abusive behavior. Investigation of child abuse potential as a dimensional marker instead of relying solely on substantiated cases offers the advantage of not only addressing the issue without underrepresentation but also proactively tackling the family-related factors that contribute to an adverse environment for the child, including the risk of abuse (16). In addition, as the CAPI does not ask directly for acts of abuse or neglect, it may have a higher acceptability among parents.

Attachment theory highlights the importance of childhood experiences with the primary caregiver in the lifelong formation of close bonds (19). Children construct internal working models of their attachment figures out of their interactions with their caregiver (20). Once organized, these internal working models are thought to be relatively resistant to change, tend to operate subconsciously and contribute to the integration of cognitive, socioemotional, and behavioral capacities that influence ongoing and future relationships, e.g., with one’s own child. Consistent with this theory, a link between ELM and insecure attachment has been reported (21–23). Parents with abusive behavior exhibit higher rates of insecure attachment patterns and childhood experiences of abuse than the general population (24). Accordingly, a recent meta-analysis of 16 studies concluded that parents with insecure attachment have a significantly higher risk of perpetrating child abuse, as indicated by official records and self-reported abuse potential (25). However, whether attachment insecurity mediates the effects of maternal ELM on the risk of child abuse remains unknown.

Attachment can be conceptualized in terms of two orthogonal dimensions: insecurity and coping strategy (26, 27). The first measures feelings of insecurity within close interpersonal relationships, whereas the second reflects whether an individual adopts an approach or avoiding behavior to cope with underlying attachment insecurity. To date, no study has explored the effects of this coping strategy among insecurely attached parents on the risk of child abuse.

The experience of ELM has also been shown to influence the development of anger-related domains. This can be attributed to the fact that anger is frequently incited by stimuli characterized as threatening and aversive (28). Additionally, it is noteworthy that the outward expression of anger typically functions as a responsive mechanism directed toward receiving better treatment or forcing an opponent to withdraw. Consequently, the current trend in research underscores the significance of anger characteristics among both adult victims of child abuse and caregivers of abused children. Herrenkohl et al. (29) revealed that individuals identified as having experienced ELM three decades earlier exhibited higher anger proneness. Meta-analysis of Stith et al. (30) revealed that parental anger/hyperreactivity is a potent risk factor for child physical abuse and neglect. Notably, given that caregivers of abused children experience and express increased anger levels, including elevated *trait anger*, *anger-in* reflecting anger suppression, and *anger-out* referring to outwardly expressed anger (31), research on specific anger characteristics in mothers with ELM may clarify the possible pathways in the cycle of abuse. Interestingly, DiLillo et al. (32) reported that maternal anger mediated the effects of a maternal childhood history of sexual abuse on abuse potential. Thus, we sought to elaborate on these findings by investigating anger characteristics (trait anger, outwardly expressed anger, and anger suppression) as potential mediators for the effect of severity of maternal ELM on child abuse potential.

The overarching objective of this study was to examine the mediating pathways involved in the intergenerational cycle of abuse. Our first aim was to assess the impact of ELM history on the potential for child abuse. We hypothesized that (1) more severe maternal ELM would be associated with a higher likelihood of child abuse potential.

Our second aim was to explore the mediating roles of attachment insecurity and anger-related factors in the impact of maternal ELM on child abuse potential. We hypothesized that (2) increased maternal attachment insecurity would mediate the effect of ELM on child abuse potential, and (3) characteristics related to anger (such as trait anger, outwardly expressed anger, and anger suppression) would also mediate the influence of ELM on child abuse potential. Additionally, we aimed to conduct an exploratory analysis within a subset of mothers who exhibited insecure attachment styles. In this analysis, we investigated the association between these mothers’ coping strategies for managing attachment insecurity, specifically focusing on approach versus avoidance behaviors, and their potential for engaging in child abuse.

Considering the established association between ELM history and mental disorders (4, 5, 33, 34), which are recognized risk factors for child maltreatment and neglect (30, 35, 36), we implemented controls to address the co-occurrence of maternal psychopathology and two other factors that could potentially influence the risk of child maltreatment: maternal years of education and partnership status (37, 38).

## 2. Materials and methods

### 2.1. Procedure

The study was performed within a multicenter project, “Understanding and Breaking the Intergenerational Cycle of Abuse”^1^ that aims to investigate the intergenerational effects of maternal experience of childhood abuse and maternal psychopathology on mother–child interaction and child well-being (39, 40). We recruited 254 mothers of children aged 5–12 years by advertisement in two German cities, Berlin and Heidelberg (flyer and poster in, e.g., pediatric, psychiatric, and gynecological outpatient clinics, public youth, or health services; recontacted participants from previous study). Following our research questions on the intergenerational effects of abuse and mental disorders of the UBICA project, the advertisement addressed mothers with a history of ELM and/or remitted major depression (rMDD) and/or borderline personality disorder (BPD) as well as healthy mothers. Due to this specific recruitment strategy, the rates of ELM, rMDD, and BPD in this sample exceeded the general population prevalence. To account for these high co-occurring mental disorders, three dichotomous variables for rMDD, BPD, and other acute axis I disorder were entered as covariates in all our analyses. We specifically recruited mothers who reported at least moderate severity of sexual or physical abuse based on the Childhood Experience of Care and Abuse (CECA) interview (41) to ensure a diverse range of abuse severity in our sample. This led to a relatively high prevalence on both of these scales in our sample (Table 1). Our analyses employed a dimensional sum score of all five CECA main scales and its severity. Notably, this study was approved by the ethics committee of the Charité—Universitätsmedizin Berlin and University Hospital Heidelberg. Upon receiving a comprehensive explanation of the procedure, all participants provided signed informed consent.

**Table 1:** Demographic and clinical characteristics.

Characteristics	Sample M(S.D.)/% (*n*)
Age	38.87 (5.79)
Years of education	16.86 (3.64)
Nationality (German)	91.7%
Partnership status	
Single	20.1% (*n* = 51)
In partnership	79.9% (*n* = 203)
Mothers with a history of moderate/severe ELM	58.6% (*n* = 149)
Sexual abuse	44.9% (*n* = 67)
Physical abuse	67.1% (*n* = 100)
Emotional abuse	23.5% (*n* = 35)
Neglect	30.9% (*n* = 46)
Parental antipathy	61.7% (*n* = 92)
Mothers with psychiatric disorders	
rMDD	55.5% (*n* = 141)
BPD	14.2% (*n* = 36)
Other	15% (*n* = 38)
Severity of cumulative ELM^*^ (range 5–20)	9.16 (3.71)
Child abuse potential (CAPI)	181.44 (41.33)
Attachment Insecurity (VASQ)	30.02 (8.37)
Mothers with insecure attachment (VASQ Insec ≥30)	47.2% (*n* = 120)
Trait anger (STAXI)	19.92 (5.72)
Anger-in (STAXI)	15.85 (4.83)
Anger-out (STAXI)	14.04 (4.06)

Anger-in, anger suppression; anger-out, outwardly expressed anger; CAPI, Child abuse potential inventory; BPD, Borderline personality disorder; ELM, Early-life maltreatment; rMDD, Major depressive disorder in remission; STAXI, The State–Trait Anger Expression Inventory; and VASQ, The vulnerable attachment questionnaire. ^*^Cumulation of ELM includes five abuse forms (neglect, physical, emotional, sexual abuses, and parental antipathy) with severity range of each form 1–4, with 4 indicating severe maltreatment.

The data were collected during two test days. During the first visit, women were interviewed with the M.I.N.I. to establish diagnoses of acute and lifetime DSM-IV axis I disorders (42). During the second visit, the Childhood Experiences of Care and Abuse (CECA) interview and the International Personality Disorder Examination (IPDE) were conducted to collect retrospective maternal experiences of abuse and to assess axis II disorders, respectively (41, 43). Between both visits, which were scheduled 1–4 weeks apart, mothers completed the questionnaires to assess attachment (the Vulnerable Attachment Style Questionnaire, VASQ), anger (the State–Trait Anger Expression Inventory, STAXI), and child abuse potential (the Child Abuse Potential Inventory, CAPI) (14, 27, 44). Besides measures used in this study, other information was collected, e.g., the affect recognition task in mothers and the assessment of mother–child interaction. Mothers received 100 EURO for participating in the study.

The exclusion criteria were conditions that may potentially affect mother’s cooperation in the study, such as lifetime history of schizophrenia, manic episodes, neurological diseases, anxious-avoidant, or antisocial personality disorder as assessed by the Mini-International Neuropsychiatric Interview (MINI) (42) and the International Personality Disorder Examination (IPDE) (43), or acute suicidality. Acute suicidality was only a temporary exclusion criterium, as we included mothers in the study, after they had stabilized. Another exclusion criterion was change of psychotropic drug dosage within 2 weeks prior to entering the study or benzodiazepine medication within the past 6 months.

### 2.2. Measures

#### 2.2.1. Early-life maltreatment

To assess the maternal experience of ELM, the German version of the CECA was implemented (41, 45). The CECA is a widely used semi-structured clinical interview that collects retrospective accounts of adverse childhood experiences, including neglect, physical or emotional abuse, antipathy from different parent figures, and sexual abuse by any perpetrator before the age of 17. It primarily focuses on objective information regarding parental behavior rather than the interviewee’s subjective feelings (41). The CECA is considered the gold standard for the retrospective assessment of childhood maltreatment (46). In this study, interviewers were psychologists holding at least a bachelor’s degree and having accomplished a 3-day training held by the author. All experiences were rated according to predetermined criteria and manualized threshold examples on four-point scales of severity (“severe,” “moderate,” “mild,” or “little/none”). Lower scores on the four-point scale typically indicate higher maltreatment severity. To ease interpretation, we re-coded these scores, with higher scores indicating higher severity. In our analyses, the sum score of all five CECA main scales with scoring range between 5 and 20 was utilized. Previous studies found reliability scores ranging from good to excellent with inter-rater reliabilities of *κ = 0*.*82* for physical abuse, *κ = 1*.*00* for sexual abuse, *κ = 0*.*98* for relationship to perpetrator and inter-respondent agreement of *κ = 0*.*77* (41).

#### 2.2.2. Child abuse potential

Abuse potential was assessed using the German version of the Child Abuse Potential Inventory (CAPI) (14) called Eltern-Belastungs-Screening zur Kindeswohlgefährdung (15). The CAPI is a widely used 63-item self-report questionnaire of adverse parental characteristics with intra- and interpersonal difficulties such as unhappiness, low self-esteem, feelings of isolation and loneliness, and unrealistic or inflexible expectations regarding children’s behavior that are associated with risk for child maltreatment. It was originally developed to assess the risk of physical abuse, although significantly increased abuse potential scores have also been found in families with other forms of abuse and neglect. Milner found in his study that the CAPI scores could be utilized to identify 81.4% of confirmed child abusers and 99% of comparison parents in a sample of 198 parents containing 43 confirmed child abusers (18). Another study supported the incremental future predictive validity of the CAPI score for official maltreatment reports (Wald = 7.0, *p* < 0.01) (17). It is important to note that the present study did not use CAPI scores to categorize parents into abusive vs. non-abusive but focused on its dimensional measure for the risk of parental abusive behavior. The CAPI contains validity indices, such as random responding and faking, which did not indicate any bias in our study sample. Internal consistency for the German version is very good (*Cronbach’s α* = 0.91) (15). Zero to 422 is the score range and scores above 207 are considered as “at high-risk” for child maltreatment.

#### 2.2.3. Attachment insecurity

Attachment insecurity was assessed using a brief self-report questionnaire: the Vulnerable Attachment Style Questionnaire (VASQ) (27). The VASQ includes 22 five-point Likert-scaled items that evaluate the degree of adult attachment vulnerability with two dimensional scores: *Insecurity* (VASQ Insec) and *Proximity-Seeking* (VASQ Proxy). The subscale *Insecurity* reflects blockages to intimacy and closeness due to fearfulness (of being hurt or let down) and hostility (feeling people are against one and anger that others have not done enough for one). The subscale *Proximity-seeking* assesses the coping strategy in terms of approach or avoidance that individuals use to manage their insecurity (i.e., some individuals with high insecurity develop excessive neediness of others, while other individuals develop an aversion to closeness with others). Low proximity seeking is characterized by avoidant behavior in interpersonal relationships, while high proximity seeking is defined as approach behavior. According to the scheme proposed here, the VASQ Proxy captures a coping strategy in terms of approach/avoidance behavior only when the level of the underlying insecurity is high (cut-off: VASQ Insec score > =30). We decided to use only the VASQ Insec score in our mediation analysis and evaluate the VASQ Proxy exploratively only among mothers with insecure attachment. VASQ has been shown to have good reliability with *Cronbach α* of 0.82 for the insecurity scale and 0.67 for proximity-seeking items. Scores range are between 12 and 60 for the insecurity and between 10 and 50 for the proximity-seeking subscale.

#### 2.2.4. Anger

Maternal trait anger and anger expression were evaluated using the German version of the State–Trait Anger Expression Inventory (STAXI) (47, 48). It is a self-report questionnaire that has been systematically developed to reflect the multidimensional nature of the anger construct: emotion (anger), hostility (trait anger), and aggression (anger expression). The trait anger scale with 10 items refers to a stable personality dimension of the tendency to experience anger. That is, high-trait anger individuals experience more frequent and more intense anger. The anger expression scale with 24 items comprises the following dimensions: anger-in, anger-out, and anger control. The anger-in subscale measures the extent to which an individual “holds things in” or suppresses anger when feeling angry, whereas the anger-out subscale evaluates the amount of anger expressed outwardly, typically in negative ways, such as cursing or throwing things. Anger control reflects one’s effort in prevention of getting angry and calming down when feeling angry. We refrained from analyzing the STAXI measure of state anger (referring to current, situational anger) and anger control (partly concerning socially desirable anger expression), as we saw less utility for our research question and aimed to focus on a personal tendency to experience and express anger (trait-anger, anger-in, and anger-out). Respondents rated each STAXI item on a four-point scale with score range between 10 and 40 for trait anger and between 8 and 24 for anger-in and anger-out. Internal consistency for the German version in clinical sample are satisfying (*Cronbach’s α* between 0.65 and 0.96) (44).

#### 2.2.5. Maternal psychopathology

To evaluate the maternal history of depression and other current DSM-IV (1994) axis I disorders, we conducted the MINI (42)—a fully structured diagnostic interview showing good interrater reliability (κ = 0.79–1.00) (49). The criteria of BPD, antisocial and anxious-avoidant personality disorder according to ICD-10 (1992) were assessed using the IPDE (43, 50)—a structured clinical interview with established reliability interrater (κ = 0.72) and test*–*retest reliability (*r* = 0.55–0.82). In our analyses, we used three dichotomous covariables for rMDD, BPD, and other acute axis I disorder.

### 2.3. Data analytic plan

To address our research questions, we conducted four mediation analyses. Maternal ELM was entered as the predictor, maternal child abuse potential as the outcome, and attachment insecurity as well as trait-anger, anger-in, and anger-out as potential mediators. In all analyses, we controlled for maternal mental disorders (three dichotomous covariables: rMDD, BPD, and other current DSM-IV axis I disorders), the mother’s years of education, and relationship status. We chose ordinary least squares regression-based path modeling (PROCESS) as we consider it the most widespread method for simple mediation analysis. Another applicable method for these analyses would be structural equation modeling (SEM). Of note, SEM and PROCESS are considered mathematically equivalent when applied to mediation models with a continuous mediator and continuous outcome variable (51). All analyses were performed in IBM SPSS Statistics Version 27 with the PROCESS v4.0 macro by Andrew F. Hayes.^2^ PROCESS is a path analysis modeling tool for estimating direct and indirect effects in mediator models. We used a single mediation path model for all mediators. Bootstrapping with 10,000 samples together with heteroscedasticity consistent standard errors (52) was employed to compute the 95% confidence intervals and inferential statistics. Effects were considered significant when the confidence interval did not include zero (53).

The data analyzed showed no indications of extreme outliers, non-normality, non-linearity, or multicollinearity. As questionnaires were missed for some individuals, data from 244 (96.1%) VASQ Insec, 251 (98.8%) trait anger, 249 (98%) anger-out, and 248 (97.6%) anger-in questionnaires were used in our analysis. Notably, the size of total effect varies between models due to different sample sizes. Bivariate Pearson and point-biserial correlations (for categorical variables like mental disorder and partnership status) were conducted to examine associations between all relevant study variables (Table 2). The explorative analysis of the VASQ Proxy was performed only among mothers with insecure attachment (VASQ Insec ≥30).

**Table 2:** Intercorrelations among key study variables.

	1	2	3	4	5	6	7	8	9	10	11	12	13
1. Severity of ELM	1												
2. Child abuse potential	0.365^**b^	1											
3. VASQ Insec	0.438^**b^	0.525^**b^	1										
4. Trait Anger	0.257^**b^	0.306^**b^	0.518^**b^	1									
5. Anger-in	0.288^**b^	0.357^**b^	0.563^**b^	0.306^**b^	1								
6. Anger-out	0.148^*b^	0.184^**b^	0.289^**b^	0.738^**b^	0.045^b^	1							
7. Years of education	−0.164^**b^	−0.218^**b^	−0.220^**b^	0.059^b^	−0.184^**b^	0.101^b^	1						
8. Maternal age	−0.111 ^b^	−0.106^b^	−0.150^*b^	−0.030^b^	−0.109^b^	−0.030^b^	0.274^**b^	1					
9. rMDD	0.283^**a^	0.335^**a^	0.444^**a^	0.228^**a^	0.305^**a^	0.155^*a^	−0.115^a^	0.009^a^	1				
10. BPD	0.290^**a^	0.307^**a^	0.427^**a^	0.409^**a^	0.314^**a^	0.330^**a^	−0.145^*a^	−0.254^**a^	0.207^**c^	1			
11. Other current psychiatric disorders	0.283^**a^	0.207^**a^	0.367^**a^	0.188^**a^	0.173^**a^	0.116^a^	−0.115^a^	−0.184^**a^	0.334^** c^	0.304^**c^	1		
12. Nationality	0.029^a^	−0.095^a^	−0.072^a^	−0.087^a^	−0.053^a^	−0.052^a^	0.008^a^	0.046 ^a^	−0.015^c^	−0.035^c^	−0.04^c^	1	
13. Partnership status	−0.053^a^	−0.168^**a^	−0.090^a^	0.024^a^	0.002^a^	0.014^a^	0.062^a^	−0.045^a^	−0.183^**c^	−0.106^c^	−0.038^c^	0.038^c^	1

Anger-in, Anger suppression; Anger-out, Outwardly expressed anger; BPD, Borderline personality disorder; ELM, Early-life maltreatment; rMDD, Major depressive disorder in remission; VASQ Insec, Attachment insecurity. ^a^Point-biserial correlation coefficient. ^b^Pearson’s *r* correlation coefficient. ^c^Phi coefficient. ^**^p < 0.01, ^*^p < 0.05.

## 3. Results

The study sample included 254 mothers, of which 199 had experienced at least one form of abuse and 149 had been abused with at least moderate severity up to the age of 17 years (see Table 1 for detailed demographics). In total, 56.3% of the mothers in our sample were diagnosed with mental disorders. Among mothers with at least moderate ELM, 40.9% met the criteria for BPD or rMDD. Intercorrelations between the study variables are displayed in Table 2.

We performed four simple mediation analyses to estimate indirect effects via attachment insecurity and anger characteristics (trait-anger, anger-in, and anger-out) for the effect of maternal ELM on child abuse potential. In each analysis, we controlled for maternal psychopathology, years of education, and partnership status. Higher maternal ELM severity was associated with increased child abuse potential, supporting our first hypothesis (total effect in all models with *β* from 2.41 to 2.67 and with *p* from 0.002 to 0.004).

### 3.1. The mediating effect of attachment insecurity

**Figure 1:**
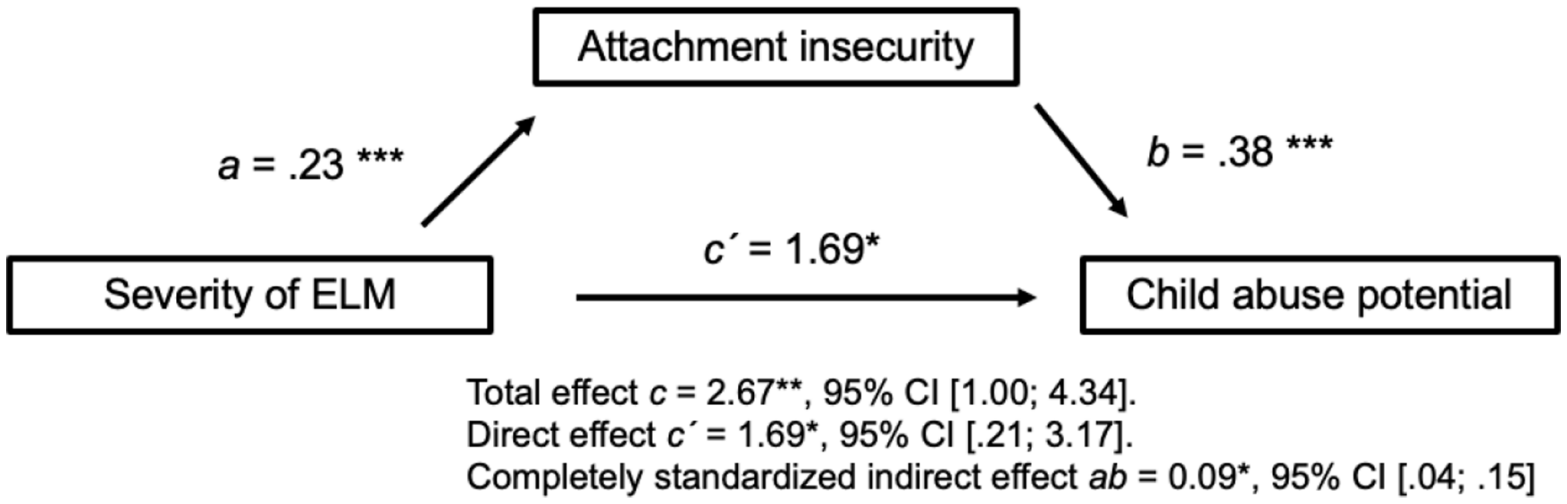
Simple mediation analysis with attachment insecurity as the mediator. Mediation is present as the confidence interval of the indirect effect *ab* excludes zero. Controlled with five covariables: rMDD, BPD, other acute axis I disorders, partnership status, and mother’s years of education. *N* = 244. ELM, Early-life maltreatment; rMDD, Major depressive disorder in remission; BPD, Borderline personality disorder. ^*^p < 0.05, ^**^p < 0.01, and ^***^p < 0.001.

We found an indirect effect of ELM on child abuse potential through attachment insecurity (indirect effect *ab* = 0.09, 95% CI [0.04; 0.15]), supporting our second hypothesis. As shown in Figure 1, higher maternal ELM predicted increased attachment insecurity (*a* = 0.23, *p* < 0.001) resulting in greater abuse potential (*b* = 0.38, *p* < 0.001). Notably, ELM influenced abuse potential independently of its effect via attachment insecurity (direct effect *c´* = 1.69, 95% CI [0.21; 3.17], *p* = 0.03) indicating a partial mediation effect.

### 3.2. The mediating effect of anger suppression

**Figure 2:**
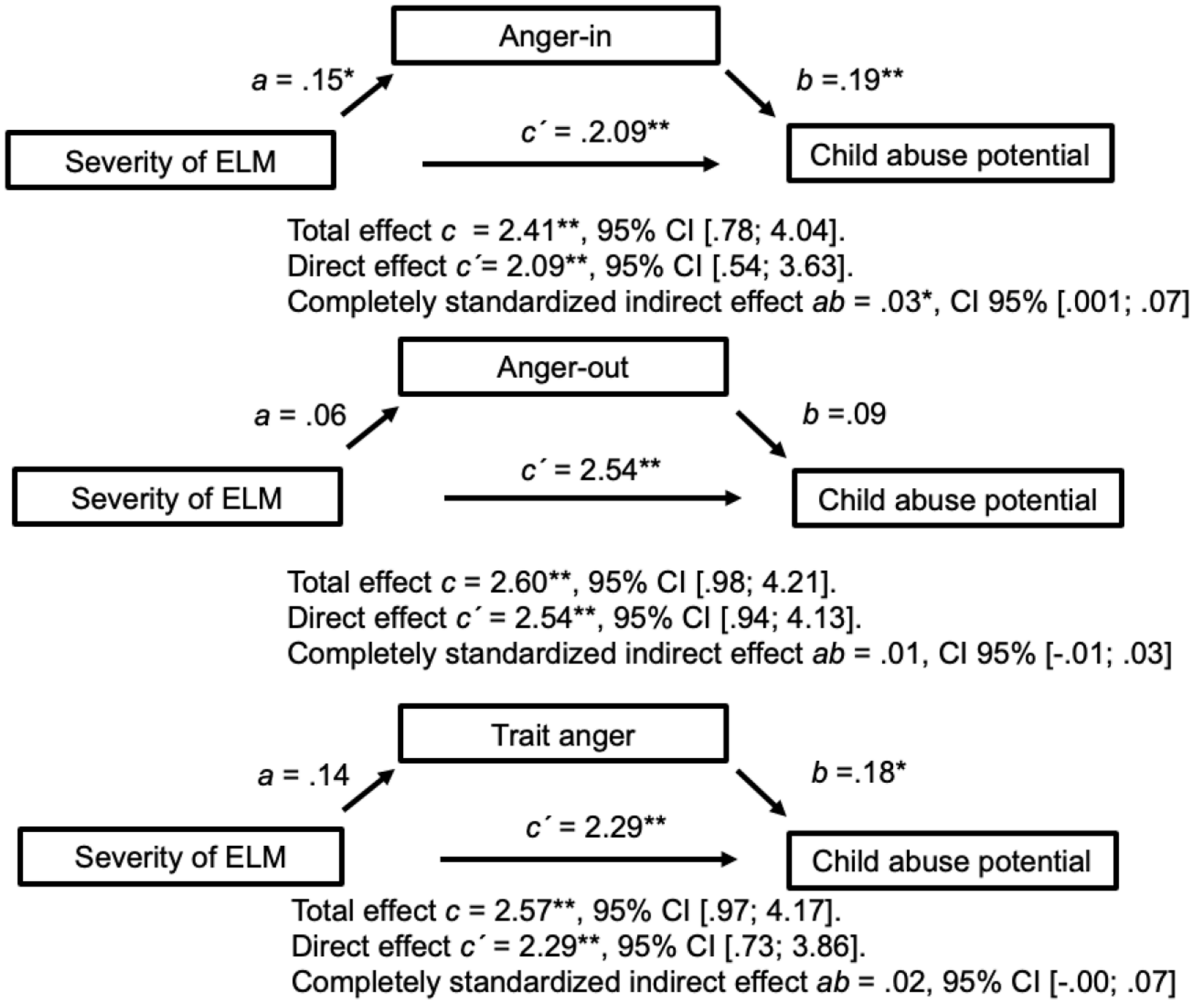
Three simple mediation analyses with anger-in, anger-out, and trait anger as mediators. Only anger-in is a significant mediator as the confidence interval of the indirect effect excludes zero. Controlled with five covariables: rMDD, BPD, other acute axis I disorders, partnership status, and mother’s years of education. *N* = 248 for anger-in; *n* = 249 for anger-out; and *n* = 251 for trait anger. Anger-in, anger suppression; anger-out, outwardly expressed anger; ELM, Early-life maltreatment; rMDD, Major depressive disorder in remission; and BPD, Borderline personality disorder. ^*^p < 0.05, ^**^p < 0.01, ^***^p < 0.001.

Early-life maltreatment also influenced abuse potential through its effect on anger-in but not anger-out or trait anger, partly supporting our third hypothesis. As shown in Figure 2, higher ELM predicted higher anger-in (*a* = 0.15, *p* = 0.03), resulting in increased abuse potential (*b* = 0.19, *p* = 0.004). ELM still influenced abuse potential independently of its effect via anger-in (direct effect *c´* = 2.09, 95% CI [0.54; 3.63], *p* = 0.009), indicating a partial mediation effect.

In contrast, maternal anger-out was not associated with ELM (*a =* 0.06*, p =* 0.44), nor did it predict abuse potential (*b =* 0.09*, p =* 0.19). Although higher trait anger predicted higher child abuse potential (*b =* 0.18, *p =* 0.01), it had no mediating effect because it was not significantly related to ELM (*a =* 0.14*, p =* 0.06).

The explorative analysis of coping strategy (VASQ Proxy) among insecurely attached mothers showed no significant correlation of proximity-seeking behavior with child abuse potential (*r* = 0.14, *p* = 0.12). Intercorrelations of proximity seeking with other variables among insecurely attached individuals are displayed in Supplementary Table S1.

Our study focused on the individual mediating effects of attachment insecurity and anger domains in the effect of ELM on child abuse potential. Nonetheless, we additionally performed a parallel mediation model with both significant mediators (i.e., attachment insecurity and anger suppression) to identify the effect of each mediator in a shared model. In this parallel mediation model, we found a significant mediating effect for attachment insecurity but not anger suppression (Supplementary Figure S2). Although the mediating effect of attachment insecurity was significantly larger, the effect of anger suppression is not negligible as it was significant in the simple mediation model.

## 4. Discussion

The present study examined maternal attachment insecurity and anger characteristics (trait anger, outwardly expressed anger, and anger suppression) as potential pathways between ELM and child abuse potential. Increased levels of insecure attachment and anger suppression were found to partially mediate the effect of maternal ELM on child abuse potential. Other characteristics of anger, such as outwardly expressed anger and trait anger, did not mediate this effect. In addition, approach or avoidant behavior as a coping strategy for insecure attachment showed no association with child abuse potential.

### 4.1. The mediating effect of attachment insecurity

Previous research has shown that ELM may lead to insecure attachment patterns and has linked attachment insecurity with abusive parenting (21–23, 25). We extended these results by showing that attachment insecurity mediates the effect of maternal ELM severity on child abuse potential, thus potentially perpetuating a cycle of abuse across generations.

Attachment insecurity reflects attitudes and feelings of discomfort with others, which can include fear of being hurt, hostility with feeling that people are against one and anger at being let down, and may result in mistrust issues (27). Our findings that higher maternal ELM predicted higher attachment insecurity corroborated the attachment theory-based explanations on how early abusive environments can contribute to the development of insecure attachment, which can persist into adulthood: a caregiver who is simultaneously a child’s source of safety but also fear by showing insensitive or even threatening behavior promotes ambivalent and negative expectations regarding the availability and trustworthiness of others; it may also lead to a negative self-image as incompetent and unworthy [for review see (20)].

These ambivalent and negative expectations can have negative impacts on mothers’ relationships with their own children by causing difficulties in understanding the children’s needs and evoking in mothers more negative feelings toward the children, such as distrust, frustration, and anger. Insecurely attached parents might also rely less on the help of others, resulting in a lack of support, feeling of isolation, and high level of stress. These factors have all been associated with a higher risk of abusive parenting (15). Negative behaviors and abusive patterns from their own childhood might be repeated due to a lack of alternative models of behavior, with unrealistic or inflexible expectations regarding the child’s behavior probably transferable. According to our results, strengthening attachment security among mothers with an ELM history could prevent the perpetuating cycle of abuse by promoting positive self-esteem, personal control, greater happiness in relationships, and better emotional management, resulting in less intra- and interpersonal stress [see review in (54)].

Notably, no associations were found between child abuse potential and approach or avoidance behavior as coping strategies in individuals with high attachment insecurity. Therefore, we conclude that the primary factor contributing to the intergenerational cycle of abuse is underlying maternal attachment insecurity rather than the coping strategy developed.

### 4.2. The mediating effect of anger suppression

We also found a second mediator for the effect of maternal ELM on child abuse potential: anger suppression. Although previous studies have reported higher anger-out and anger proneness in individuals with childhood maltreatment (29, 55), we found only anger suppression (anger-in) to be associated with ELM. This disparity probably occurred because Herrenkohl et al. (29) did not consider co-occurrent psychopathology and Win et al. (55) considered only a certain range of mental disorders. Notably, mental disorders are a frequent sequela of ELM and have been linked with aggression, anger proneness, and an increased risk of child maltreatment (4, 5, 30, 34, 35, 56, 57).

As anger suppression also predicted child abuse potential, a significant mediation effect emerged. Anger suppression might be an adaptive strategy for individuals with an ELM history to avoid conflicts, although it might also lead to higher unresolved anger. In very stressful contexts or close and intense interactions, like those of mother and child, suppressed anger may be acted out, resulting in a higher risk for child maltreatment.

Corroborating findings by Plate et al. (31), we also observed higher trait anger to predict higher child abuse potential, probably because mothers with higher anger proneness show an increased susceptibility to their children’s misbehavior, resulting in more parent–child conflicts. However, the mediation did not reach significance, because ELM severity did not predict trait anger.

The mediating effect of anger suppression in the parallel mediation model with attachment insecurity was not significant. This result may be explained through the moderately-high association between anger suppression and attachment insecurity in our study sample, which is in accordance with previous empirical studies (10, 11) and theoretical considerations (9). Thus, strengthening attachment security may result in reduction of anger suppression and conversely, reducing anger suppression may strengthen attachment security. However, as our study focuses on reducing the intergenerational risk for child abuse and both attachment insecurity and anger suppression were significant mediators in the simple mediation model, we encourage to address both in prevention measures.

### 4.3. Limitations

Possible limitations to consider when interpreting our findings are as follows: First, we only assessed mothers as they are still often the primary caregiver and mothers acting alone perpetrate almost 40% of child abuse (58). Therefore, future studies should include fathers to acknowledge the paternal impact and discriminate potential differences between mothers and fathers concerning the mediating role of attachment and anger. Especially for anger, differences may exist between mothers and fathers, as women tend to expect greater social costs of anger expression and to suppress anger in unequal relationship contexts (59). Secondly, we chose to examine child abuse potential as a dimensional risk marker instead of relying on substantiated cases of child abuse. This approach addresses the concern that relying exclusively on substantiated cases may result in an underestimation of the issue since not all abusive behaviors are reported to or identified by authorities (12, 13). Additionally, it emphasizes the significance of risk factors that accumulate to pose a higher risk for child abuse, which holds greater importance for preventive measures. Third, because our cross-sectional study design does not allow for conclusions on causal relations between our study variables, future research may employ longitudinal research designs to clarify causal directions. Fourth, we used self-report measures to estimate anger characteristics, attachment insecurity, and child abuse potential. To deepen the understanding of attachment and anger in the cycle of abuse, interviews or observational measures are needed. Fifth, our recruitment strategy targeted mothers with a history of ELM and/or rMDD and/or BPD. As a result, the prevalence rates of rMDD and BPD in combination with ELM in our sample may differ from general population. While we controlled for these mental health variables to minimize their potential influence on our results, we acknowledge that our findings may not be representative for the general population and, thus, need to be considered preliminary. Future research is needed to replicate our study in a more representative sample to enhance the generalizability and external validity of our findings.

### 4.4. Clinical implication

The present study contributes to the literature in that it shows that both attachment insecurity and anger suppression act as mediators of the effect of maternal ELM on child abuse potential. Importantly, this mediating role occurs independently of maternal mental disorders (we accounted for maternal psychopathology in our analysis), which are common sequelae of ELM and by themselves pose a risk for child maltreatment (4, 5, 30, 35). These findings may have several clinical implications: First, public education should emphasize attachment insecurity and anger suppression as risk factors for the intergenerational transmission of abusive behavior. Second, reaching out to parents with history of ELM, also outside the mental health system, e.g., in schools, pediatric and gynecological outpatient clinics, may be crucial for timely identification of burdened families with elevated risk for child abuse prior to the actual abuse occurring. Third, providing parental trainings targeting the identified mediators could be beneficial in supporting parents at risk for abusive behavior and in preventing actual acts of abuse. Such training could include elements capable of (a) increasing the awareness of attachment insecurity, (b) increasing the awareness of anger suppression, (c) strengthening attachment security, e.g., through metallization-based parent training, and (d) improve anger management (60–63). Fourth, as high child abuse potential might indicate that familial or parental distress is at a level that might already impair child well-being even though actual acts of abuse have not taken place (16), also non-abusive families could benefit from interventions that reduce the child abuse potential through improving parental attachment and reducing anger suppression.

### 4.5. Conclusion

Our study indicates that maternal attachment insecurity and anger suppression mediate the effect of maternal history of ELM on child abuse potential, potentially serving as pathways in the intergenerational cycle of abuse. To prevent the intergenerational transmission of child abuse, screening for parents at risk, providing general education about risk factors such as attachment insecurity and anger suppression, and offering targeted interventions that address these issues are crucial. As these associations persist even when accounting for often co-occurring risk factors such as maternal mental disorders, our results highlight the importance of addressing parents already connected to mental health care as well as those who seem personally resilient for psychiatric disorders but may still be at risk for abusive behavior.

## Data availability statement

The raw data supporting the conclusions of this article will be made available by the authors, without undue reservation.

## Ethics statement

The studies involving humans were approved by the ethics committee of the Charité—Universitätsmedizin Berlin and the ethics committee of University Hospital Heidelberg. The studies were conducted in accordance with the local legislation and institutional requirements. The participants provided their written informed consent to participate in this study.

## Author contributions

KW: Formal analysis, Writing – original draft, Visualization, Writing – review & editing. FB: Conceptualization, Funding acquisition, Investigation, Methodology, Project administration, Resources, Supervision, Validation, Writing – review & editing. KB: Conceptualization, Funding acquisition, Investigation, Methodology, Project administration, Resources, Supervision, Validation, Writing – review & editing. CH: Conceptualization, Data curation, Investigation, Methodology, Project administration, Writing – review & editing. DK: Conceptualization, Data curation, Investigation, Methodology, Project administration, Writing – review & editing. NS: Conceptualization, Data curation, Investigation, Methodology, Project administration, Writing – review & editing. AF: Conceptualization, Data curation, Investigation, Methodology, Project administration, Writing – review & editing. CN: Conceptualization, Data curation, Investigation, Methodology, Project administration, Writing – review & editing. SH: Conceptualization, Funding acquisition, Investigation, Methodology, Project administration, Resources, Supervision, Validation, Writing – review & editing. RB: Conceptualization, Funding acquisition, Investigation, Methodology, Project administration, Resources, Supervision, Validation, Writing – review & editing. SW: Conceptualization, Funding acquisition, Investigation, Methodology, Project administration, Resources, Supervision, Validation, Writing – review & editing. MK: Conceptualization, Funding acquisition, Investigation, Methodology, Project administration, Resources, Supervision, Validation, Writing – review & editing. CJ: Conceptualization, Data curation, Investigation, Methodology, Project administration, Writing – review & editing. KD: Conceptualization, Data curation, Formal analysis, Investigation, Methodology, Project administration, Writing – review & editing.

## Footnotes

^1^www.ubica.de

^2^www.processmacro.org
