# Supplementary material for: The mediating role of attachment and anger: exploring the impact of maternal early-life maltreatment on child abuse potential
Source: Front Psychiatry. 2023 Oct 27;14:1267038. doi: 10.3389/fpsyt.2023.1267038 (PMC10641504; doi:10.3389/fpsyt.2023.1267038)
Supplement: Supplementary file 1 [file Data_Sheet_1.docx]

Supplementary Material

Supplementary Table S1. *Intercorrelations of proximity seeking among key study variables in insecure attached mothers.*

|  | 1 | 2 | 3 | 4 | 5 | 6 | 7 | 8 | 9 | 10 | 11 |
| --- | --- | --- | --- | --- | --- | --- | --- | --- | --- | --- | --- |
| 1. VASQ Proximity seeking | 1 |  |  |  |  |  |  |  |  |  |  |
| 2. Child abuse potential | 0,142 ^b^ | 1 |  |  |  |  |  |  |  |  |  |
| 3. Severity of ELM | 0,1 ^b^ | ,296^**b^ | 1 |  |  |  |  |  |  |  |  |
| 4. BPD | ,281^**a^ | ,192^*a^ | ,208^*a^ | 1 |  |  |  |  |  |  |  |
| 5. rMDD | 0,078 ^a^ | 0,124 ^a^ | 0,131 ^a^ | 0,12 ^b^ | 1 |  |  |  |  |  |  |
| 6. Other current psychiatric disorders | 0,129 ^a^ | 0,058 ^a^ | ,183^*a^ | ,219^*c^ | ,287^**c^ | 1 |  |  |  |  |  |
| 7. Trait Anger | ,273^**b^ | 0,176 ^b^ | 0,056 ^b^ | ,417^**a^ | 0,08 ^a^ | 0,047 ^a^ | 1 |  |  |  |  |
| 8. Anger-out | ,194^*b^ | 0,117 ^b^ | 0,077 ^b^ | ,321^**a^ | 0,099 ^a^ | 0,025 ^a^ | ,719^**b^ | 1 |  |  |  |
| 9. Anger-in | ,213^*b^ | ,277^**b^ | ,203^*b^ | ,333^**a^ | 0,093 ^a^ | 0,028 ^a^ | 0,182 ^b^ | -0,115 ^b^ | 1 |  |  |
| 10. Years of education | -,232^*b^ | -0,125 ^b^ | -,262^**b^ | -0,045 ^a^ | 0,02 ^a^ | -0,061 ^a^ | 0,086 ^b^ | 0,12 ^b^ | -0,138 ^b^ | 1 |  |
| 11. Partnership status | 0,056 ^a^ | -0,131 ^a^ | 0,054 ^a^ | -0,079 ^b^ | -0,087 ^c^ | 0,097 ^c^ | 0,111 ^a^ | 0,038 ^a^ | 0,084 ^a^ | 0,09 ^a^ | 1 |

Anger-in, anger suppression; anger-out, outwardly expressed anger; BPD, borderline personality disorder; ELM, early-life maltreatment; rMDD, major depressive disorder in remission,

a. Point-biserial correlation coefficient.

b. Pearson’s r correlation coefficient.

c. Phi coefficient.

** p< .01, *p< .05


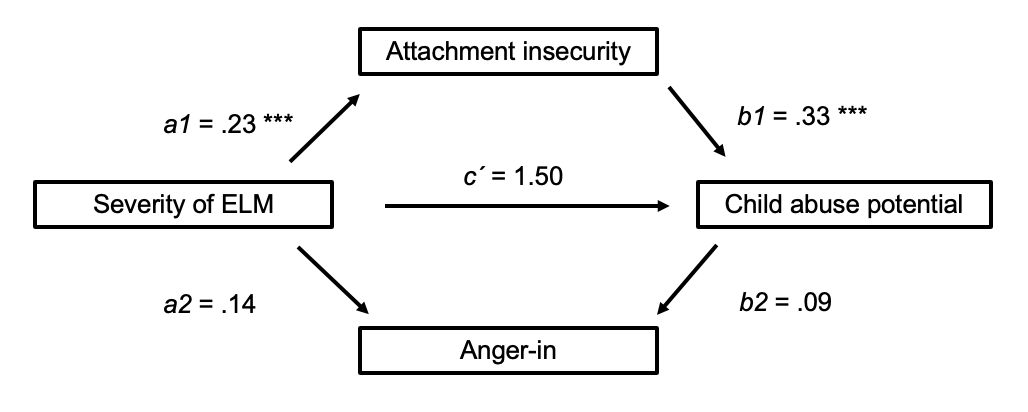


## Supplementary Figure S2. *Parallel mediation path model with attachment insecurity and anger suppression as mediators.* Significant

## mediating effect only for attachment insecurity. Total effect c = 2.48, CI 95 % [.77; 4.20], *p* = .005; direct effect c´= 1.50, CI 95 % [-.01;

## 3.01], *p* = .051; completely standardized indirect effect *ab1* = 0.08, CI 95% [.03; .13]; *ab2* = 0.01, CI 95% [-.01; .04]. Controlled with five

## covariables: rMDD, BPD, other acute axis I disorders, partnership status, and mother’s years of education. N= 239. ELM, early-life

## maltreatment; rMDD, major depressive disorder in remission; BPD, borderline personality disorder.

## * p < .05, ** p < .01, *** p < .001.
